# Assessing fatigue in children and adolescents: Psychometric validation of the German version of the PROMIS^®^ Pediatric Short Form v2.0 - Fatigue 10a in school children and pediatric chronic pain patients

**DOI:** 10.1007/s11136-021-03032-8

**Published:** 2021-11-13

**Authors:** Ariane Sommer, Susanne Grothus, Kamila Grochowska, Benedikt B. Claus, Lorin Stahlschmidt, Julia Wager

**Affiliations:** 1 German Paediatric Pain Centre, Children’s and Adolescents’ Hospital, Dr.-Friedrich-Steiner-Str. 5, 45711 Datteln, Germany; 2 Department of Children’s Pain Therapy and Paediatric Palliative Care, Faculty of Health, School of Medicine, Witten/Herdecke University, 58448 Witten, Germany; 3 PedScience Research Institute, 45711 Datteln, Germany

**Keywords:** Children and adolescents, Chronic pain, Fatigue, PROMIS^®^, Questionnaire, Validation

## Abstract

**Purpose:**

Fatigue is a common symptom in children and adolescents. Its negative impact on health outcomes is even more pronounced in those with chronic pain. There is currently no fatigue measurement tool in German that is validated for both children and adolescents with and without chronic pain. Therefore, this study aimed to gather quantitative validity evidence to support the use of the German version of the PROMIS^®^ Pediatric Short Form v2.0 - Fatigue 10a (PROMIS^®^ F-SF) in the German pediatric general population as well as in German pediatric chronic pain patients.

**Methods:**

The 10-item self-assessment questionnaire was validated in a sample of *N* = 1348 school children (9–18 years; 52.4% female) and *N* = 114 pediatric chronic pain patients (8–17 years; 63.3% female). Construct and convergent validity, reliability, and item and scale characteristics were examined.

**Results:**

Confirmatory factor analyses showed sufficient model fit for the 1-factor model of the questionnaire (school sample: CFI = 0.94, RMSEA = 0.10, SRMR = 0.04; patient sample: CFI = 0.90, RMSEA = 0.14, SRMR = 0.05). Convergent validity was supported by weak-to-large significant correlations with sleep quality, health-related quality of life (HRQoL), and pain characteristics. The questionnaire had excellent internal consistency in both samples (*α* = 0.92 and *α* = 0.93). Sex differences and age distributions of the PROMIS^®^ F-SF showed that girls reported significantly higher fatigue than boys and that fatigue increased with age.

**Conclusion:**

The PROMIS^®^ F-SF is a reliable instrument with good psychometric properties. Preliminary evidence is provided that the questionnaire validly measures fatigue in children and adolescents with and without chronic pain.

**Supplementary Information:**

The online version contains supplementary material available at 10.1007/s11136-021-03032-8.

## Introduction

Fatigue exhibits itself in symptoms like intense tiredness, lack of energy, and a feeling of profound weakness and exhaustion that can manifest mentally or physically [1–3]. The prevalence of severe fatigue in the general pediatric population is high, ranging from one-fifth to about one-third [4–8]. There are considerable sex and age differences in the occurrence of fatigue. Research shows that 20% of adolescent girls suffer from severe fatigue compared to only 6% of adolescent boys [5]. Regarding age differences, 11% of children aged 11–14 years and 17% of those aged 13–16 years suffer from severe fatigue [4].

Fatigue has a negative impact on health outcomes in children and adolescents including sleep disturbances, impaired social relationships, school absence, anxiety, depression, and poorer quality of life [3, 5]. The negative effects of fatigue are not only seen in healthy children, children with pre-existing health problems, such as recurrent pain, also struggle with the impact of fatigue on their daily life, including sleep disturbances and reduced HRQoL [3, 9–16]. Previous research also shows that fatigue negatively impacts pain characteristics such as pain intensity [12, 16], functional disability [16], and pain-related school absenteeism [11, 16, 17].

Thus, the assessment and monitoring of fatigue is critical to treatment decisions and should be used in routine care of pediatric chronic pain patients [11, 18, 19] as well as in routine or preventive application in the general pediatric population. For this, a reliable and valid measurement tool is required.

As part of the Patient-Reported Outcomes Measurement Information System (PROMIS^®^; www.nihpromis.org), funded by the National Institutes of Health (NIH), the Pediatric Short Form v2.0 - Fatigue 10a (PROMIS^®^ F-SF) [20, 21] was developed. The English fixed-length short form and items from the underlying pediatric fatigue item bank have been used in several studies including children and adolescents suffering from chronic pain [16], cancer [22, 23], and sickle cell disease [24]. The questionnaire proved to be a feasible and valid measure [16, 22] and demonstrated sensitivity to change [25]. A German version [26] of the short form already exists but has not yet been validated for healthy children and adolescents nor for those with chronic pain.

Therefore, the aim of this study was to provide quantitative validity evidence for the use of the German version of the PROMIS^®^ F-SF in a general population sample (school children) as well as in a pediatric chronic pain patient sample. It was hypothesized that the factor structure of the instrument yields good model fit in the conduction of confirmatory factor analyses (CFA). Good psychometric properties were expected. Convergent validity was assessed via associations with sleep quality, HRQoL, and pain characteristics; moderate to strong correlations were expected. In addition, in the context of convergent validity, sample differences were tested; pediatric chronic pain patients were expected to have higher fatigue scores than students. Additionally, age and gender distributions of the questionnaire were explored. Fatigue was expected to increase with age and girls were expected to experience more fatigue symptoms than boys.

## Materials and methods

### Participants and study procedure

#### School sample

As part of the MeMaps project (“Chronische Schmerzen bei Kindern und Jugendlichen – multidimensionales Ergebnisqualitätsmaß und praxistaugliche Stratifizierungsstrategie”), data were collected between October and November 2019 at three secondary schools of diverse performance level in Germany. The MeMaps project is a prospective longitudinal study with three measurement points that collects data on chronic pain in school children. For this validation study, data from the first measurement point (*N* = 1348) were used. The Ethics Committee of the University Witten/Herdecke, Germany, approved the MeMaps project (Approval code 75/2019).

Students in grades 5–11 and their parents were informed orally about the study and received written information. Students were included in the study if they had an adequate understanding of the German language and provided a consent form (filled out by the parents) as well as an assent form (filled out by the student). The PROMIS^®^ F-SF was completed by all students on electronic tablets during normal school hours as part of the broader survey battery of the MeMaps project. As all questions in the digital survey battery were mandatory, there were no missing values of the PROMIS^®^ F-SF and all 1348 datasets were included in the study analyses. *N* = 742 students experienced pain in the past four weeks, of which *n* = 419 met the definition of chronic pain, that is the presence of at least weekly recurrent pain that began at least three months ago and has been present for the past four weeks.

#### Patient sample

Data were collected at the outpatient department of the German Paediatric Pain Centre (GPPC) from June to September 2019. The German Pain Questionnaire for Children and Adolescents [27], which is part of standard evaluation at the GPPC, was filled out by patients and their parents before they visited the GPPC. Patients were all classified as chronic pain patients, as this is the prerequisite for admission to the pain clinic. The PROMIS^®^ F-SF was completed by all patients aged 8–17 years during the waiting time of their initial appointment at the GPPC. Inclusion criteria for participation in this study were: an adequate understanding of German language, a completed PROMIS^®^ F-SF, and informed parental consent to use the data for research purposes. The study was approved by the Ethics Committee of the Children’s and Adolescents’ Hospital Datteln, Germany (Approval code 2019/07/10/JW1). Altogether, the questionnaire was filled out by 140 patients. Permission to use data for research purposes was not provided by *n* = 5 patients, and a further *n* = 21 patients were excluded due to missing data in the PROMIS^®^ F-SF. *N* = 114 patient datasets were included in the analyses of this study.

Table 1 shows sample characteristics of the school and patient sample.

**Table﻿ 1:** Demographics and pain characteristics of the two study samples

		School sample				Patient sample			
		*n* ^a^	%	*M*	*SD*	*n* ^a^	%	*M*	*SD*
Sex	Male	642	47.6	–	–	42	36.8	–	–
	Female	706	52.4			72	63.2		
Age (years)		1348	–	12.83	1.95	114	–	13.61	2.65
Born in Germany		1348	95.2			111	94.7		
Pain in the past four weeks		742	55.0			114	100		
Chronic pain		419	31.1			114	100		
Pain characteristics									
Mean pain intensity ^b^		742	–	5.08	2.14	112	–	5.98	1.77
Pain-related school absence ^c^		742		0.72	2.00	88		2.57	3.84
Pain-related disability ^d^		742		22.99	9.09	112		34.79	9.67
Main pain location ^e^		742				114			
Head		420	42.4	–	–	66	57.9	–	–
Abdomen		292	29.5			12	10.5		
Back/Extremities		507	51.2			40	35.1		
Other		60	6.1			6	5.3		

*M* = Mean; *SD* = Standard deviation. ^a^*n* varies due to missing values. ^b^Numerical Rating Scale, Range 0–10. ^c^Days in the past four weeks, Range 0–20 days. ^d^Pediatric Pain Disability Index [28], Range 12–60. ^e^More than one main pain location possible

### Measures

#### Fatigue

The PROMIS^®^ Pediatric Short Form v2.0 - Fatigue 10a (PROMIS^®^ F-SF) is a self-administered instrument that measures a child's fatigue during the past week [20, 21]. It was developed for children and adolescents aged 8–17 years and consists of 10 items (e.g., “I was too tired to enjoy the things I like to do”) rated on a 5-point Likert scale ranging from 1 = never to 5 = almost always. All items load on one latent factor; therefore, a total sum score is calculated with higher scores indicating greater fatigue. Raw sum scores can be converted into standardized T-scores, calibrated in the U.S. general pediatric population, with a mean of 50 and a standard deviation of 10 [29]. A German version of the PROMIS^®^ F-SF exists [26] that has not yet been validated. The German translation is the result of a process of forward–backward translation, multiple expert reviews, cross-linguistic harmonization, and cognitive debriefing with a sample of native German speakers, a process performed for all translations of PROMIS^®^ instruments (for more information on PROMIS^®^ instruments see www.healthmeasures.net) [30].

#### Sleep quality

The German version [31] of the revised Adolescent Sleep–Wake Scale (rASWS) [32] is a self-assessment questionnaire to evaluate sleep quality and behavioral characteristics of sleep over the past month. It consists of seven items distributed among three scales: Going to Bed (two items, e.g., “When it’s time to go to bed, I want to stay up and do other things”), Reinitiating Sleep (three items, e.g., “After waking up during the night, I have trouble going back to sleep”), and Returning to Wakefulness (two items, e.g., “In the morning, I wake up feeling rested and alert”). All items have Likert scale responses from 1 = always to 6 = never with higher scores indicating better sleep quality. Internal consistency of the questionnaire ranges from acceptable to good in both samples of the current study (school sample: Cronbach’s α = 0.73 to 0.86; patient sample: Cronbach’s α = 0.71 to 0.89).

#### Health-related quality of life

HRQoL was only surveyed in the school sample using the German version of the KIDSCREEN-10 Index [33] recording HRQoL in the last week. The 10-item self-assessment questionnaire is rated on a 5-point Likert scale (1 = never/not at all, 5 = always/extremely) with higher scores indicating better HRQoL. The instrument showed good internal consistency in the school sample (Cronbach’s α = 0.86).

#### Pain characteristics

Within the questionnaire battery of the MeMaps project, pain characteristics were collected from students using the German Pain Questionnaire for Children and Adolescents [27]. As the questions regarding pain characteristics refer to a period of the past four weeks, these questions were only presented to students who reported having had pain in the past four weeks (*n* = 742).

In the patient sample, as part of the standard evaluation of the GPPC, pain characteristics were recorded using the German Pain Questionnaire for Children and Adolescents available for child and youth self-report as well as parent proxy-report [27]. Patients under 11 years of age (*n* = 20) self-reported pain locations and pain intensity measured with a faces pain scale. Therefore, for this study, data on children’s pain characteristics (except pain location) were retrieved from the parent proxy-report (*n* = 20). For patients older than 11 years, all pain characteristics (mean pain intensity, pain-related school absence, pain-related disability) were self-reported. For this study, missing youth data were replaced by parental reports (replaced data: pain intensity: 3.2%, pain-related school absence: 16.0%, pain-related disability: 4.3%). Substitution is justified by previous studies that reported strong agreement between patient and parent reports of pain characteristics [34, 35].

##### Mean pain intensity

A Numerical Rating Scale (NRS) ranging from 0 = no pain to 10 = strongest pain was utilized for the measurement of mean pain intensity over the past four weeks. It has been widely supported that the NRS is a valid and widely used instrument for recording pain intensity in children and adolescents [36].

##### Pain-related school absence

Students reported pain-related school absence as the number of days missed due to pain over the past four weeks. As is standard practice, patients were asked for pain-related school absence as the number of days missed due to pain over the past three months. To increase comparability of both samples, the number of absent school days in the patient sample was divided by 3 to obtain an estimate of the number of missed school days in the past four weeks.

##### Pain-related disability

The Pediatric Pain Disability Index (PPDI) [28], a self-report questionnaire consisting of 12 items rated on a 5-point Likert scale (1 = never, 5 = always), was used to assess pain-related disability in daily activities. Higher impairment is indicated by a higher sum of scores [28]. In the present study, the PPDI shows good to excellent internal consistency as demonstrated by Cronbach’s *α* = 0.90 in the school sample and Cronbach’s α = 0.85 in the patient sample.

### Statistical Analyses

Statistical analyses were performed using the IBM Statistical Package for Social Sciences (SPSS) version 27 for Windows. AMOS version 25 was used to conduct CFA. For all analyses significance level was set to *α* = 0.05. Correlation coefficients are interpreted as small (|*τ*|= 0.1), moderate (|*τ*|= 0.3), or large (|*τ*|= 0.5) [37]. For all analyses, the raw data of the fatigue measurement were used.

#### Construct validity

For both samples, CFA were conducted to examine the factor structure of the fatigue measurement. A 1-factor model was assumed, and Maximum-Likelihood was used as the estimation method. The model fit was assessed by two recommendations. The first was conservative [38]: *χ*^2^/*df* (≤ 3 = acceptable, ≤ 2 = good), Comparative Fit Index (CFI; ≥ 0.95 = acceptable, ≥ 0.97 = good), Root Mean Square Error of Approximation (RMSEA; ≤ 0.08 = acceptable, ≤ 0.05 = good), and Standardized Root Mean Square Residual (SRMR; ≤ 0.10 = acceptable, ≤ 0.05 = good). The second was more general [39], where a better model fit is indicated by smaller values of *χ*^2^/*df* (e.g., < 2), Comparative Fit Index (CFI) values closer to 1.0, Root Mean Square Error of Approximation (RMSEA), and Standardized Root Mean Square Residual (SRMR) values closer to 0.0.

#### Item and scale characteristics

Cronbach’s alpha (α) was calculated to evaluate the internal consistency of the PROMIS^®^ F-SF. Item ranges, means, and standard deviations as well as corrected item-total correlations of the measure were computed. In addition, scale means and standard deviations as well as T-scores were reported.

#### Convergent validity

Correlations between fatigue, sleep quality, HRQoL and pain characteristics were calculated using Kendall’s Tau. For the school sample, correlations between fatigue and pain characteristics were performed only for students suffering from pain in the past four weeks (*n* = 742). Correlations between fatigue and HRQoL were examined only for students. Independent *t* tests were carried out to check whether the two samples differed in their experiences of fatigue.

#### Sex and age distribution of the PROMIS^®^ F-SF

Sex differences in the PROMIS^®^ F-SF were assessed using independent *t* tests and Kendall’s Tau correlations between age and fatigue.

## Results

### Construct validity

The overall model fits of the 1-factor model of the PROMIS^®^ F-SF for the school sample and the patient sample are presented in Table 2. The model fit of the patient sample was slightly worse than the one of the school sample [39]. According to conservative recommendations [38], all but one fit index - the SRMR - were not within the acceptable range. In both samples, all items showed high factor loadings (see Table A1).

**Table 2:** Model fit indices of the 1-factor model of the PROMIS® F-SF for both samples

	*χ*^2^	*df*	*p*	*χ*^2^/*df*	CFI ^a^	RMSEA ^b^	RMSEA ^b^ 90-% CI		SRMR ^c^
School sample	492.0	35	< 0.001	14.1	0.94	0.10	0.09	0.11	0.04
Patient sample	110.8	35	< 0.001	3.2	0.90	0.14	0.11	0.17	0.05

^a ^Comparative Fit Index. ^b ^Root Mean Square Error of Approximation. ^c ^Standardized Root Mean Square Residual

### Item and scale characteristics

#### School sample

The PROMIS^®^ F-SF showed excellent internal consistency with Cronbach’s α = 0.92. The average total score among students was *M* = 19.27 (*SD* = 8.18) which corresponds to a T-value of 47.9 (reference group: U.S. general pediatric population).

#### Patient sample

Internal consistency of the PROMIS^®^ F-SF was excellent with Cronbach’s α = 0.93. The average total score was *M* = 20.96 (*SD* = 8.99) which converts to a T-value of 49.1 (reference group: U.S. general pediatric population).

Table 3 displays item properties of the PROMIS^®^ F-SF for both samples.

**Table 3:** Item properties of the German version of the PROMIS^®^ F-SF for both samples

Item	School sample				Patient sample			
	*M*	*SD*	Range	corrected item–total correlations	*M*	*SD*	Range	corrected item–total correlations
1	2.04	1.08	1–5	0.73	2.05	1.25	1–5	0.68
2	1.67	1.00	1–5	0.68	1.85	1.16	1–5	0.68
3	2.17	1.16	1–5	0.71	2.55	1.19	1–5	0.74
4	2.51	1.24	1–5	0.66	2.71	1.34	1–5	0.74
5	1.86	1.07	1–5	0.77	1.88	1.01	1–5	0.77
6	1.84	0.98	1–5	0.74	1.91	0.95	1–5	0.66
7	2.15	1.14	1–5	0.75	2.28	1.24	1–5	0.78
8	1.68	0.98	1–5	0.68	2.03	1.19	1–5	0.73
9	1.71	0.97	1–5	0.70	1.93	1.05	1–5	0.75
10	1.63	0.97	1–5	0.70	2.05	1.25	1–4	0.80

*M* = Mean, *SD* = Standard deviation

### Convergent validity

Significant weak to moderate correlations were found between the PROMIS^®^ F-SF and the three scales of the sleep questionnaire in the school sample. In the patient sample, significant moderate correlations were found between the PROMIS^®^ F-SF and the scales Reinitiating Sleep and Returning to Wakefulness. Table 4 provides an overview of the correlations between the PROMIS^®^ F-SF and the rASWS scales.

**Table 4:** Correlations of the PROMIS^®^ F-SF and the rASWS scales for both samples

PROMIS® F-SF		Going to Bed		Reinitiating Sleep		Returning to Wakefulness	
	*n* ^a^	*τ*	*p*	*τ*	*p*	*τ*	*p*
School sample	1348	− 0.18	< 0.001	− 0.31	0.010	− 0.36	< 0.001
Patient sample	103	− 0.06	0.388	− 0.42	< 0.001	− 0.36	< 0.001

^a ^*n* varies due to missing values of the rASWS in the patient sample

Correlation analyses in the school sample showed a strong significant association between fatigue and HRQoL (*τ* =  − 0.51, *p* < 0.001).

Table 5 displays the correlations between fatigue and pain characteristics in both samples. In the school sample, all pain characteristics were significantly correlated with fatigue showing weak to moderate associations. For the patient sample, a weak significant association was found between fatigue and pain-related disability.

**Table 5:** Correlations of the PROMIS^®^ F-SF and pain characteristics

PROMIS® F-SF	Mean pain intensity ^a^			Pain-related school absence ^b^			Pain-related disability ^c^		
	*n* ^d^	*τ*	*p*	*n* ^d^	*τ*	*p*	*n* ^d^	*τ*	***p***
School sample	742	0.17	< 0.001	742	0.08	0.010	742	0.41	< 0.001
Patient sample	112	0.08	0.274	88	0.01	0.857	112	0.18	0.006

^a ^Numerical Rating Scale, Range 0–10. ^b ^Days in the past four weeks, Range 0–20 days. ^c ^Pediatric Pain Disability Index, Range 12–60 [28]. ^d ^*n* varies due to missing values in the patient sample

An independent *t* test indicated that the patient sample (*M* = 20.96, *SD* = 8.99) had higher fatigue values compared to the school sample (*M* = 19.27, *SD* = 8.18), (*t*(129.34) =  − 1.94, *p* = 0.055, *d* =  − 0.21). Students who suffered from pain in the past four weeks (*M* = 22.06, *SD* = 8.57) reported significantly higher fatigue scores than students without pain in the past four weeks (*M* = 15.85, *SD* = 6.15), (*t*(1324.56) = 15.46, *p* < 0.001, *d* = 0.82).

### Sex and age distributions of the PROMIS^®^ F-SF

Female students (*M* = 21.38, *SD* = 8.98) showed significantly higher fatigue scores than male students (*M* = 16.95, *SD* = 6.46), (*t*(1280.24) =  − 10.45, *p* < 0.001, *d* =  − 0.56). The same was found for the patient sample; an independent *t* test showed that girls (*M* = 22.89, *SD* = 9.33) reported significantly higher fatigue than boys (*M* = 17.64, *SD* = 7.35), (*t*(102.16) =  − 3.32, *p* < 0.001, *d* =  − 0.61). Age was significantly associated with fatigue for students (*τ* = 0.22, *p* < 0.001) as well as for patients (*τ* = 0.16, *p* = 0.022) showing increasing fatigue with rising age.

## Discussion

The aim of this study was to validate the German version of the PROMIS^®^ F-SF for both school children and pediatric chronic pain patients. CFA showed sufficient model fit for the 1-factor model. Convergent validity was supported by significant weak to strong correlations with sleep quality, HRQoL, and pain characteristics, as well as by higher fatigue scores in children with pain than in those without pain. The questionnaire had excellent internal consistency in both samples. Girls generally reported higher fatigue scores than boys and fatigue increased with age.

### Construct validity

CFA revealed a slightly better model fit in the school sample than in the patient sample [39]. When interpreting fit indices according to conservative recommendations [38], all but one fit index - the SRMR - were not within the acceptable range. However, it should be noted that in both samples all items had high factor loadings, which indicates strong associations between the items and the factor. Furthermore, internal consistency of the questionnaire is excellent. Due to these additional psychometric characteristics and considering international comparability of questionnaire results, the model was maintained in its original form despite the limited model fit.

### Item and scale characteristics

Excellent internal consistency confirms that the fatigue questionnaire is a reliable instrument and underlines the good suitability of the instrument for use in the German pediatric community as well as in the population of pediatric chronic pain patients. In previous research, the English short version of the fatigue instrument has shown sensitivity to change [25]; this should be assessed for the German version in future research.

In general, the fatigue scores in both German samples were rather low compared to the previous research [5, 16]. On average, both students and patients reported the occurrence of fatigue symptoms as “almost never.” T-scores were just below the mean of 50, suggesting that German children tended to have lower fatigue scores compared to the U.S. general pediatric population. This should be considered when interpreting T-scores based on the U.S. reference population.

In contrast to previous research [4, 5], over 60% of students in the current study “almost never” experienced fatigue symptoms in the past seven days. Contrary to this, a clinical review summarized that about one-third of European children experience considerable fatigue symptoms four times a week or more often [8]. As the mentioned studies used different instruments to assess and determine the prevalence of fatigue, a comparison with the present study is difficult.

Unexpectedly, pediatric chronic pain patients in the present study reported a rather low frequency of fatigue symptoms. Only less than 2% of patients reported fatigue symptoms “often” to “almost always.” In a previous study, using the English version of the PROMIS^®^ F-SF, pediatric chronic pain patients had T-scores on average 10 points higher than those of the patients in the present study [16].

Regardless, low fatigue scores were evident in both students and patients with chronic pain. This may indicate a general cultural difference in fatigue between German samples and other European or American samples. To our knowledge, there have been no comparative international studies on the severity of fatigue in children and adolescents with and without chronic pain. The validation of the German version of the PROMIS^®^ F-SF and the large variety of translations of the PROMIS^®^ instrument could enable such studies and create comparability through the standardization of measurement instruments.

### Convergent validity

The associations between sleep quality and fatigue were all in expected directions, indicating that the more fatigue symptoms were experienced, the worse the quality of sleep. This was evident in both samples, with the strength of the association lower than expected, ranging from weak to moderate. The two scales Reinitiating Sleep and Returning to Wakefulness were moderately associated with the fatigue assessment. Surprisingly, the Going to Bed scale was only weakly associated with the fatigue questionnaire. However, these results support the convergent validity of the fatigue measure given the moderate association with two out of three subscales.

Further evidence of convergent validity is the large negative association between fatigue and HRQoL in the school sample, indicating that the more fatigue symptoms were experienced, the worse the HRQoL. This is in line with the previous research showing that fatigue worsens HRQoL especially in children with long-term conditions (for example chronic pain) [3, 11].

Convergent validity was also inspected using pain characteristics. These findings were not in line with expectations as the strength of associations was lower than anticipated. In both samples pain-related disability had the strongest association, followed by pain intensity and pain-related school absence. The latter two were not significant in the patient sample and only weak in the school sample. A similar weak correlation (*r* = 0.27, *p* < 0.001) between pain intensity and fatigue was found in a study of pediatric chronic pain patients using the English version of the PROMIS^®^ F-SF [16]. In another study of patients with juvenile idiopathic arthritis and juvenile dermatomyositis, parents' ratings of worst pain were moderately correlated with fatigue (*r* = 0.51, *p* < 0.001) [12]. Additionally, there is some research that attributes fatigue as having an important role in school functioning [17]. For instance, fatigue is considered to be a predictor of school functioning in pediatric chronic pain patients [16] and a mediator between pain and school functioning [11]. To our knowledge, the direct association between school absence and fatigue has not yet been investigated and needs further research attention, especially in pediatric chronic pain patients. Overall, the associations between fatigue and pain characteristics in the school sample, albeit weaker than expected, provide preliminary support for convergent validity of the questionnaire (especially the association with pain-related disability). However, the extremely weak associations of fatigue with pain intensity and pain-related school absence in the patient sample were surprising. Further research is needed to better understand these associations and whether results will be replicated with other pain measures.

As a final aspect of convergent validity, it was hypothesized that pediatric chronic pain patients would have higher fatigue scores than students. While this was true, there was no significant difference between the two samples. Regardless, there was a significant difference between students with pain and students without pain; students who reported pain in the past four weeks had significantly higher fatigue scores than students who did not report pain. Even more surprising was that students with pain showed higher fatigue scores than pediatric chronic pain patients.

Overall, results of this study provide preliminary support for convergent validity of the German version of the fatigue questionnaire.

### Sex and age distributions of the PROMIS^®^ F-SF

There was a sex difference for fatigue in both study samples. In line with the previous research [5, 24], girls in tertiary care as well as in the general population reported significantly higher fatigue scores than boys. However, there is one study using the English version of the PROMIS^®^ F-SF that has not found a significant sex difference in fatigue [16].

In accordance with the previous research [4, 16], age had a significant positive association with fatigue in both study samples, indicating that the older the participants were, the more they experienced fatigue symptoms. In contrast, another study found no significant association between fatigue and age, however, the participants in that study were young adults between 15 and 30 years [24]. The increase in fatigue symptoms with age may be specific to younger children.

### Limitations

The presented study has several limitations that need to be considered when interpreting the results. It should be noted that pain characteristic instruments used to assess convergent validity had different reference periods than the fatigue instrument (fatigue questionnaire: last seven days, pain-related instruments: last four weeks). Further research is needed to better understand whether results will be replicated with other pain measures. In addition, the patient sample self-reports of certain pain characteristics had to be partially replaced by parent proxy-reports. Despite the proven high agreement between parent and child reports for pain characteristics [34, 35], this may have weakened the associations between measures. Last, patient sample is relatively small. Psychometric studies that use larger or other clinical samples are needed to further support the findings presented here.

## Conclusion

This study shows that the German version of the PROMIS^®^ F-SF is a reliable instrument with good psychometric properties. It provides preliminary support that the measure is valid for assessing fatigue in children and adolescents with and without chronic pain. The multiple translations of the PROMIS^®^ instrument allow international comparisons and further research regarding fatigue. Therefore, this study contributes to the improvement of international research in the field of fatigue.

## Supplementary Information

Below is the link to the electronic supplementary material.

Supplementary file1 (DOCX 16 kb)

## Data Availability

Not applicable.
