# Supplementary material for: Assessing fatigue in children and adolescents: Psychometric validation of the German version of the PROMIS® Pediatric Short Form v2.0 - Fatigue 10a in school children and pediatric chronic pain patients
Source: Qual Life Res. 2021 Nov 13;31(4):1257–66. doi: 10.1007/s11136-021-03032-8 (PMC8960656; doi:10.1007/s11136-021-03032-8)
Supplement: Supplementary file 1 — Supplementary file1 (DOCX 16 kb) [file 11136_2021_3032_MOESM1_ESM.docx]

**Appendix**

Table A1. Factor loadings of the all items of the 1-factor CFA model for both samples.

| **Items** | **1** | **2** | **3** | **4** | **5** | **6** | **7** | **8** | **9** | **10** |
| --- | --- | --- | --- | --- | --- | --- | --- | --- | --- | --- |
| **School sample** | .77 | .71 | .74 | .69 | .80 | .78 | .79 | .70 | .73 | .73 |
| **Patient sample** | .69 | .70 | .76 | .76 | .81 | .70 | .81 | .76 | .79 | .83 |
